# H-Ferritin-nanocaged olaparib: a promising choice for both BRCA-mutated and sporadic triple negative breast cancer

**DOI:** 10.1038/s41598-017-07617-7

**Published:** 2017-08-08

**Authors:** S. Mazzucchelli, M. Truffi, F. Baccarini, M. Beretta, L. Sorrentino, M. Bellini, M. A. Rizzuto, R. Ottria, A. Ravelli, P. Ciuffreda, D. Prosperi, F. Corsi

**Affiliations:** 1 Dipartimento di Scienze Biomediche e Cliniche “Luigi Sacco”, Università degli Studi di Milano, Milano, Italy; 2 Dipartimento di Biotecnologie e Bioscienze, Università degli Studi di Milano-Bicocca, Milano, Italy; 3 Dipartimento di Scienze Biomediche, Chirurgiche ed Odontoiatriche, Sezione di Tossicologia Forense, Università degli Studi di Milano, Milano, Italy; 4 Unità di chirurgia generale ad indirizzo senologico, Istituti Clinici Scientifici ICS Maugeri Pavia Spa SB, Pavia, Italy

## Abstract

Poly(ADP-ribose) polymerase (PARP) inhibitors represent a promising strategy toward the treatment of triple-negative breast cancer (TNBC), which is often associated to genomic instability and/or BRCA mutations. However, clinical outcome is controversial and no benefits have been demonstrated in *wild type* BRCA cancers, possibly due to poor drug bioavailability and low nuclear delivery. In the attempt to overcome these limitations, we have developed H-Ferritin nanoformulated olaparib (HOla) and assessed its anticancer efficacy on both BRCA-mutated and non-mutated TNBC cells. We exploited the natural tumor targeting of H-Ferritin, which is mediated by the transferrin receptor-1 (TfR1), and its physiological tropism toward cell nucleus. TNBC cell lines over-expressing TfR-1 were successfully recognized by H-Ferritin, displaying a fast internalization into the cells. HOla induced remarkable cytotoxic effect in cancer cells, exhibiting 1000-fold higher anticancer activity compared to free olaparib (Ola). Accordingly, HOla treatment enhanced PARP-1 cleavage, DNA double strand breaks and Ola delivery into the nuclear compartment. Our findings suggest that H-Ferritin nanoformulation strongly enhances cytotoxic efficacy of Ola as a stand-alone therapy in both BRCA-mutated and *wild type* TNBC cells, by promoting targeted nuclear delivery.

## Introduction

In the era of tailored medicine, breast cancer (BC) is often successfully treated by targeted therapy^1^. Hormonal and anti-HER2 therapies are the treatment of choice for luminal BC and HER2-positive BC, respectively^1^. However, targeted therapy is not available for triple-negative breast cancer (TNBC), a BC subtype associated to poor clinical outcome and frequent local and distant recurrence. Therefore, combinatorial cytotoxic chemotherapy remains the recommended option for TNBC treatment^2–4^. In recent years, the interest of clinicians has moved toward poly(ADP-ribose) polymerase (PARP) inhibitors, which act by causing impairment of one of the main mechanisms of DNA repair, i.e. the base excision repair (BER)^5^. PARP inhibitors offer a promising therapeutic strategy for cancers that are deficient in Breast Related Cancer Antigens (BRCA) 1 and/or 2 and that have lost the homologous recombination (HR) mechanism of DNA repair regulated by BRCA-1 and 2 genes^6, 7^. HR is used as long as the BER and the nucleotide-excision repair (NER) have failed. Therefore, a concept of “synthetic lethality” has been suggested, in which it was established that the treatment of BRCA-deficient cancers with PARP inhibitors deprives BC cells of both BER and HR repair mechanisms, resulting in the arrest of the cell cycle with subsequent cell death^8^. Since a significant proportion of TNBCs exhibits defects in HR mechanism, the BRCA-like character of TNBC, so called “BRCAness”, has been explored and exploited as a possible therapeutic target^9^.

Among PARP inhibitors, olaparib (Ola, AZD 2281, AstraZeneca, London, UK) has been assessed in chemotherapy regimens for BRCA-mutated or HR-deficient breast and ovarian cancer, and several clinical trials are ongoing^10, 11^. However, issues regarding its clinical potential have been raised. Indeed, whereas Ola displayed great anticancer activity toward high-grade serous or poorly differentiated ovarian cancer, globally controversial results have been obtained with TNBC, demonstrating a certain benefit only in BRCA-mutated BCs. Recently, a clinical trial comparing Ola treatment in BRCA-mutated and sporadic TNBCs failed in showing positive response in both cases^12^. This result was somehow unexpected, considering that up to 24% of *wild type* (wt) BRCA ovarian cancers had previously proved to be responsive to PARP inhibitors. The controversial effect of Ola in TNBC suggested that different reasons beyond BRCA status could be involved in the therapeutic outcome of Ola. First, Ola exhibited poor bioavailability and required a daily dosage of 800 mg/kg to achieve anticancer efficacy. Current formulations of the drug only achieve sub-optimal plasma exposure of Ola, and, as a result, the amount of drug able to reach the tumor and to enter malignant cells is lower than expected^13, 14^. In addition, TNBC cells can develop resistance to Ola due to the overexpression of multidrug resistance proteins, such as P-glycoprotein (P-gp) and Breast Cancer Resistance Protein (BCRP)^15^. Thus, we reasoned that enhancing Ola bioavailability and tumor delivery could have strongly improved Ola efficacy in TNBC, even beyond BRCA status. Nanotechnology offers smart solutions to overcome the major challenges of bioavailability and targeted delivery of oncological drugs through targeted nanosystems^16, 17^. Among them, protein based-nanocages represent an exciting solution^18^. In particular, H-ferritin nanoparticles, consisting of a 24-mer of self-assembled human ferritin H-chain (HFn), hold great promise, since they combine low toxicity with high stability in biological fluids, they could be easily loaded with drugs and be modified by surface chemistry or genetic engineering^19^. HFn is specifically recognized by the transferrin receptor-1 (TfR1), which is over-expressed in several human cancer subtypes, including TNBC^20^, and promotes the cellular internalization of these nanoparticles. HFn nanocages were demonstrated to be able to mediate the direct delivery of cytotoxic doxorubicin into the nuclear compartment of cancer cells through a self-triggered mechanism activated by enhanced ROS production^21, 22^. In addition, the metronomic administration of doxorubicin-HFn nanodrug in a 4T1 mouse model of metastatic BC demonstrated potent antitumor efficacy associated with inhibition of angiogenesis, reduced chemoresistance and negligible cardiotoxicity^23^. With the present study, we aimed to develop a novel HFn-based nanoformulation of Ola (HOla) able to achieve tumor targeting, improve cellular uptake of the drug and its nuclear release. This nanocarrier was expected to greatly improve Ola anticancer efficacy in both BRCA-mutated and wt TNBC cells.

## Results

### Suitability of H-Ferritin for Ola nuclear targeting in TNBC cells

**Figure 1:**
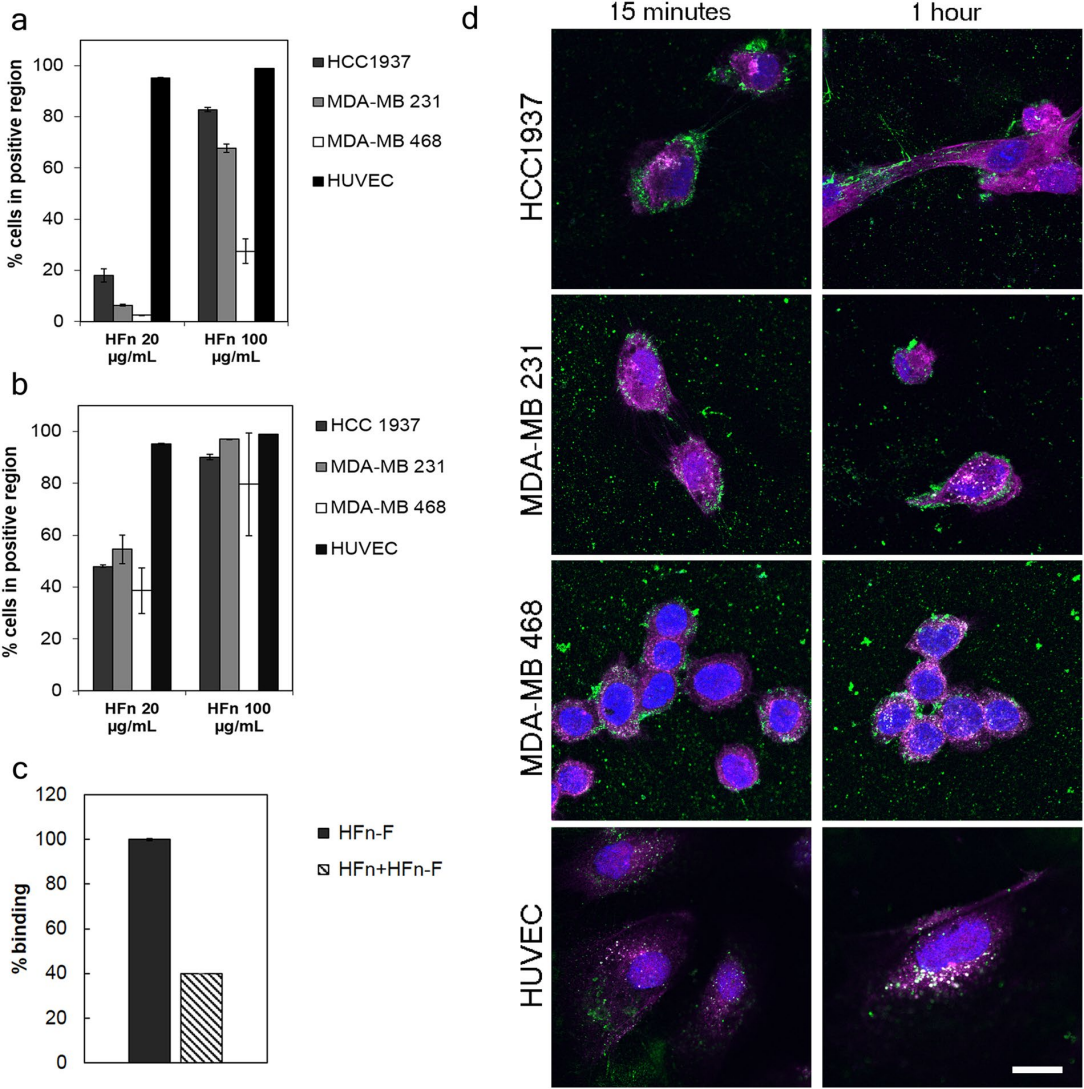
HFn recognition of Triple-Negative Breast Cancer cells. (**a**) MDA-MB 231, MDA-MB 468, HCC1937 (for TNBC) and HUVEC cells were incubated 2 h at 4 °C in PBS buffer and 0.3% BSA with different amounts of FITC-labelled HFn nanoparticles (20 and 100 µg/mL). Then, cells were processed for flow cytometry, using untreated cells to set the positive region and the singlet gate. Reported values are the mean ± s.e. (n = 3). (**b**) MDA-MB 231, MDA-MB 468, HCC1937 (for TNBC) and HUVEC cells seeded in a multiwell plate were incubated 1 h at 37 °C in complete cell culture medium with different amounts of FITC-labeled HFn nanoparticles (20 and 100 µg/mL). Then, cells were detached, washed and processed for flow cytometry, using untreated cells to set the positive region and the singlet gate. Reported values are the mean ± s.e. (n = 3). (**c**) Competition assay. HCC1937 cells were incubated 1 h at 37 °C with 100 µg/mL of FITC-labelled HFn with or without an excess of unlabeled HFn (*i.e*., 5 mg) as competitor. Cells were then detached and treated for flow cytometry. Untreated cells have been used to set the singlet gate and the positive region. Reported values are the mean ± s.e. (n = 3). (**d**) Colocalization of HFn and TfR1. Confocal images of MDA-MB 231, MDA-MB 468, HCC1937 (for TNBC) and HUVEC cells incubated 15 min or 1 h at 37 °C in complete cell culture medium with FITC-labeled HFn nanoparticles (green; 100 µg/mL). Nuclei were stained with DAPI (blue). TfR1 was recognized with anti-TfR1 antibody (Abcam) and labeled with an anti-rabbit secondary antibody conjugated with Alexa Fluor 546 (magenta; Thermo Fischer Scientific). Scale bar: 10 µm.

HFn nanocages were assessed for their capability to interact with TNBC cells, using MDA-MB 231 (PTEN wt, p53 mutant, BRCA1 wt), MDA-MB 468 (PTEN-null, p53 mutant and BRCA1 wt) and HCC1937 (PTEN mutant, p53-null, BRCA1 mutant) cell lines. TNBC cells were first analyzed for membrane expression of TfR1 (Supplementary Fig. 1a) and total amount of cytosolic ferritin heavy chain (Supplementary Fig. 1b). Human Umbilical Vein Endothelial Cells (HUVEC) with high TfR1 expression were selected as control cells to determine the overall safety of HOla in healthy cells. TfR1 was found over-expressed in all of the selected cell lines, with HCC1937 and MDA-MB 468 displaying a 0.5–0.6-fold higher expression compared to MDA-MB 231, whereas the amount of endogenous ferritin heavy chain was similar in all the tested cell lines. The binding assays, performed by flow cytometry after incubating the cells with 20 or 100 µg/mL of FITC-labeled HFn, demonstrated a good and dose-dependent recognition of all the TNBC cell lines both at 4 °C and 37 °C (Fig. 1a and b). HFn bound to MDA-MB 468 to a lesser extent, despite the high TfR1 expression, but nevertheless the binding remained dose-dependent. Increased percentage of FITC-positive cells was observed upon binding at 37 °C, which could be probably attributable to endocytic mechanisms (Fig. 1b). Competition assay performed on HCC1937 cells confirmed HFn specific interaction with TfR1-positive cells, as displayed by the 60% reduction in cell binding when an excess of unlabeled HFn was added as a competitor (Fig. 1c). The occurrence of HFn-TfR1 interaction was further confirmed by confocal microscopy at short incubation times (15 min and 1 h)(Fig. 1d). Confocal laser scanning microscopy images of TNBC cells incubated with FITC-labeled HFn revealed a fast internalization of the nanoparticles in all the tested cell lines. Indeed, HFn were recovered attached to the cell membrane after only 15 min of incubation, while they were found almost completely internalized after 1–3 h. In addition, the fluorescence signal of HFn dramatically dropped at 48 h of incubation (Supplementary Fig. 2), in accordance with results from previous studies^22, 23^. HFn internalization route was investigated by assessing the colocalization with specific markers of endocytic compartments, i.e. EEA1 for early endosome, GM130 for the Golgi apparatus, CatD for lysosomes, Tf for recycling endosomes and LAMP-1 for late endosomes. As previously observed with other cancer cell lines, HFn accumulated in early endosome at 1 and 3 h. The absence of colocalization with markers of Golgi, late and recycling endosomes corroborated the hypothesis that HFn neither is pushed out of the cells through recycling endosomes nor is forwarded to the Golgi *via* the late endosomes. Moreover, HFn exclusion from the lysosomes suggested that the nanocages did not undergo lysosomal degradation within the time window explored (Supplementary Fig. 3).

**Figure 2:**
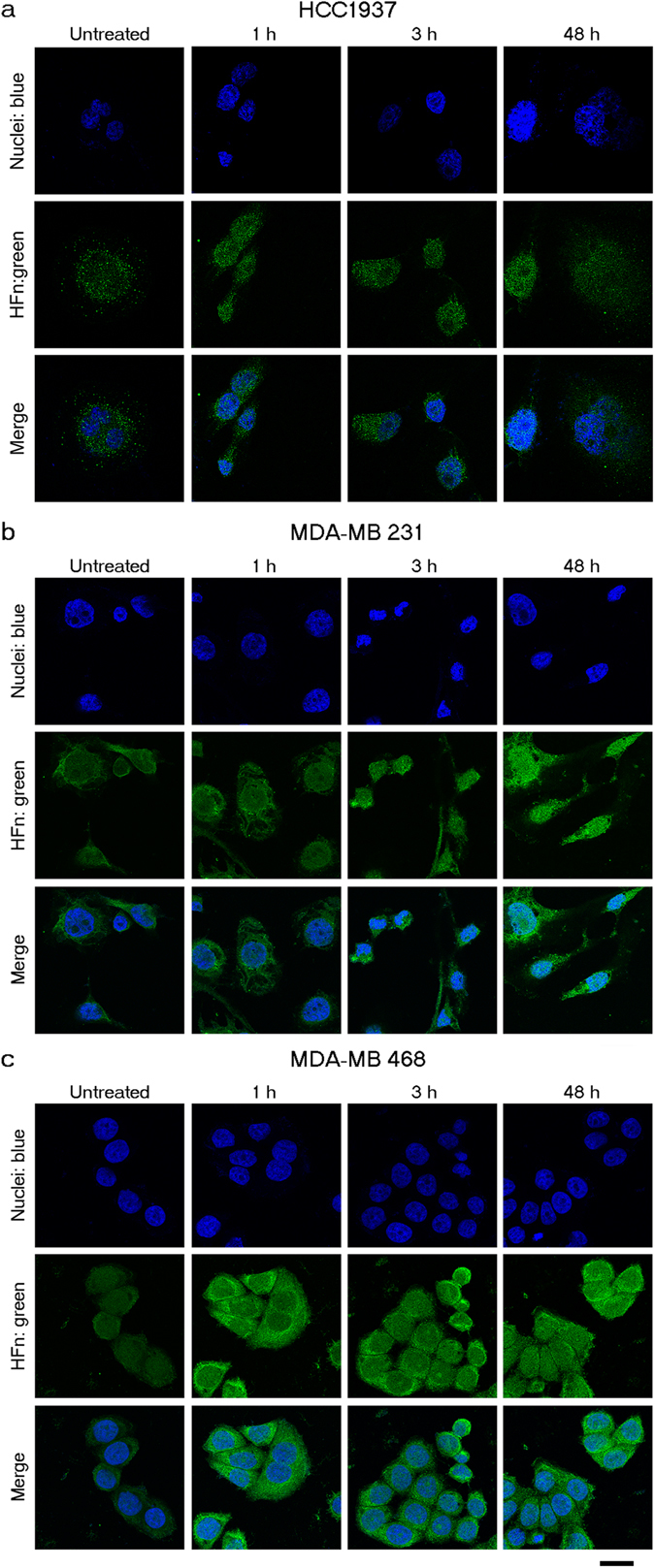
Treatment with Ola triggers nuclear translocation of endogenous HFn. Confocal microscopy images of MDA-MB 231, MDA-MB 468 and HCC1937 cells incubated for 1, 3 and 48 h at 37 °C with 1 µM Ola. Cells not previously treated with Ola were used as negative control (untreated). Nuclei were stained with DAPI (blue). Endogenous ferritin was recognized with anti-ferritin antibody and labeled with an anti-rabbit secondary antibody conjugated with Alexa Fluor 488 (green; Thermo Fischer Scientific). Scale bar: 10 µm.

Our previous studies suggested that HFn could be an ideal nanovector to trigger drugs’ nuclear delivery, by following the HFn nuclear translocation upon noxious stimuli^21, 22^. Cell stimulation with doxorubicin activates the intracellular ROS production, induces the massive nuclear translocation of HFn, and promotes the subsequent release of the cargo inside the nucleus. Although Ola specifically inhibits PARP-1, an enzyme translocated into the nuclear compartment concurrently to protein translation^24^, there is no evidence of its capability to induce HFn nuclear translocation. Therefore, we assessed the effect of Ola treatment on the localization of endogenous ferritin. Confocal microscopy images of HCC1937, MDA-MB 231 and MDA-MB 468 cells treated with 1 µM Ola exhibited a time-dependent accumulation of endogenous ferritin inside the nuclei, similar to what was observed with doxorubicin (Fig. 2)^22, 23^. We concluded that HFn properties could be suitable for Ola delivery.

### Development of HFn-Ola nanodrug

**Figure 3:**
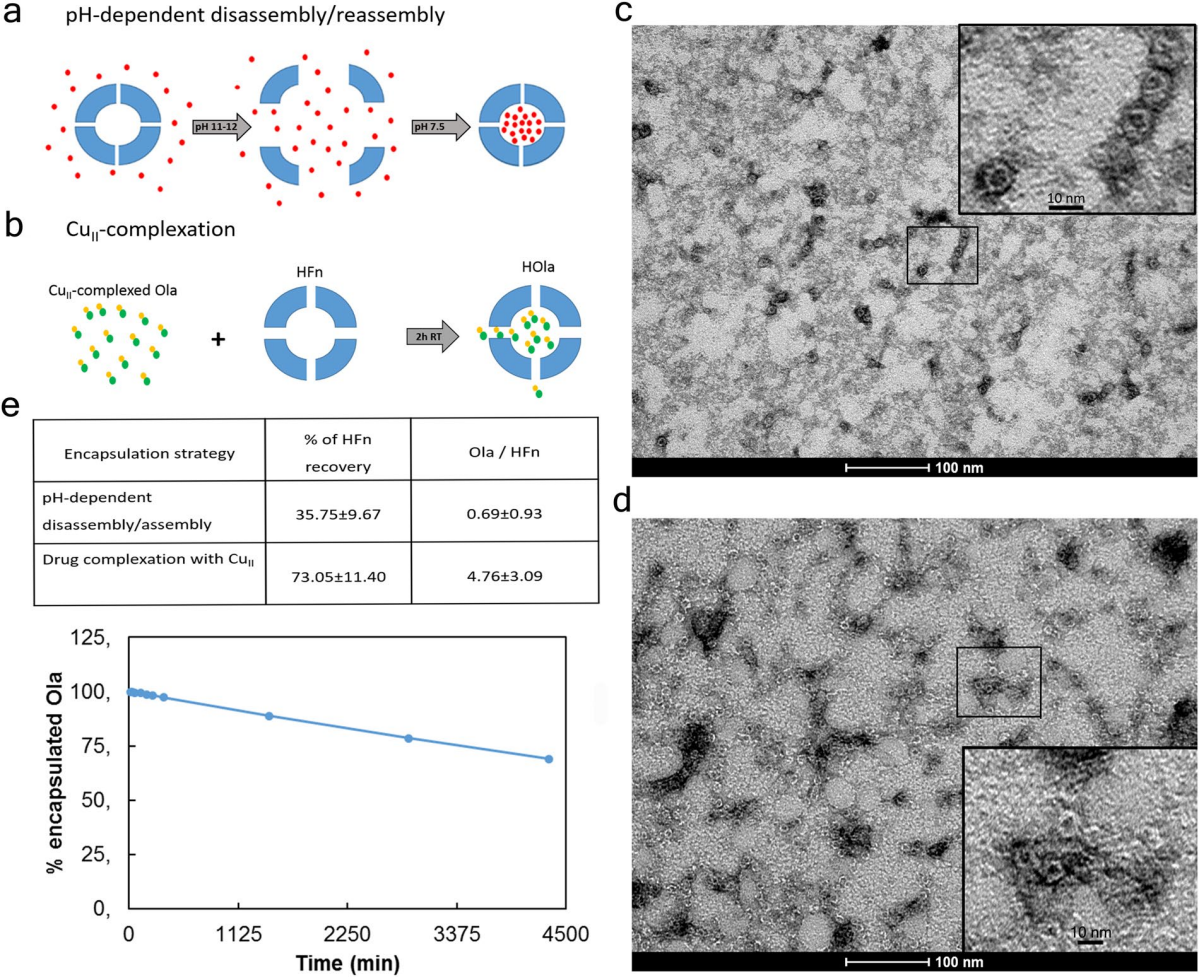
Development and characterization of HOla. (**a**) Schematic representation of the pH-dependent disassembly/reassembly method used for HFn loading with Olaparib. Olaparib is represented in red. (**b**) Schematic representation of the HFn loading strategy through Olaparib pre-complexation with Cu(II). Olaparib is represented in green, while Cu(II) in yellow. (**c**) Electron microscopy images of Olaparib-loaded HFn nanoparticles obtained with the pH-dependent disassembly/reassembly strategy or (**d**) with the Cu(II) pre-complexation. (**e**) Summary of yields of HFn encapsulation with Olaparib. (**f**) Drug release from HOla nanoparticles. The kinetic of Ola release from HFn nanocages was determined by HPLC-MS quantifying the amount of Ola released in the dialysis medium. The nanodrug is highly stable at 37 °C in PBS buffer. The release from HFn nanocage is less than 3% even after 6 h incubation.

To set up the experimental conditions for Ola loading in HFn, two different procedures were explored. In a first attempt, we followed a modification of the pH-dependent disassembly/reassembly method already exploited for different cargo molecules (Fig. 3a)^22^. In detail, HFn shell disassembled into monomers at alkaline pH (around 11–12). Simultaneously, hydrophobic Ola was solubilized by sonication in 0.1 M NaOH, and added to the mixture of disassembled HFn monomers. Next, the pH was mildly brought to neutrality to allow the complete refolding of HFn cage, which naturally encloses Ola molecules. In a second strategy, the HFn capability to drive the uptake of metal ions was considered^25^. Ola was complexed with Cu(II) by incubation with 10 mM CuSO_4_. Then, the complexed drug was added to HFn and incubated to allow Cu(II)-Ola to be incorporated in HFn as depicted in Fig. 3b. HOla was separated by free Ola through gel filtration, and the amount of recovered HFn and Ola was determined by Bradford assay and by mass spectrometry, respectively. HOla nanocages obtained from both procedures were analyzed by transmission electron microscopy (TEM): HOla obtained by Cu(II)-mediated loading strategy showed cave sphere structures, thus demonstrating that the quaternary structure was intact and correctly folded (Fig. 3c); by contrast, low-defined structures were detected upon the pH-dependent loading procedure (Fig. 3d). Moreover, results reported in Fig. 3e pointed out that the high hydrophobicity of Ola strongly reduced HOla yields, probably affecting its capability to accurately refold into the quaternary structure after disassembly. In contrast, the Cu(II)-based strategy afforded 7-fold increase in loading yields, with significantly improved HOla nanocage stability in solution. HOla obtained with the Cu(II)-based strategy were characterized by Dynamic Light scattering (DLS) to assess their hydrodynamic size and the ζ-potential after loading. No significant alterations in HFn size and charge were found after Ola loading. Size of void HFn and HOla were 10.7 ± 2.5 nm and 14.3 ± 4.2 nm, respectively, while ζ-potential was −24.8 ± 0.8 mV and −20.5 ± 1.2 mV. These results suggested that Ola was essentially incorporated inside the nanocage and that no, or limited, residual adsorption on protein surface remained after sample isolation.

Next, Ola release from HFn was analyzed *in vitro*. The release kinetics was assessed by dialyzing HOla at 37 °C in phosphate buffer saline (PBS), pH 7.2, and measuring Ola leakage from HFn by quantitative mass spectrometry in the supernatants. The amount of encapsulated Ola remained constant during the first 6 h of incubation in PBS, then decreased by 30% at 72 h of incubation (Fig. 3f), confirming adequate stability of the nanoformulation at physiological pH.

### HOla exhibits enhanced antiproliferative activity in cancer cells

**Figure 4:**
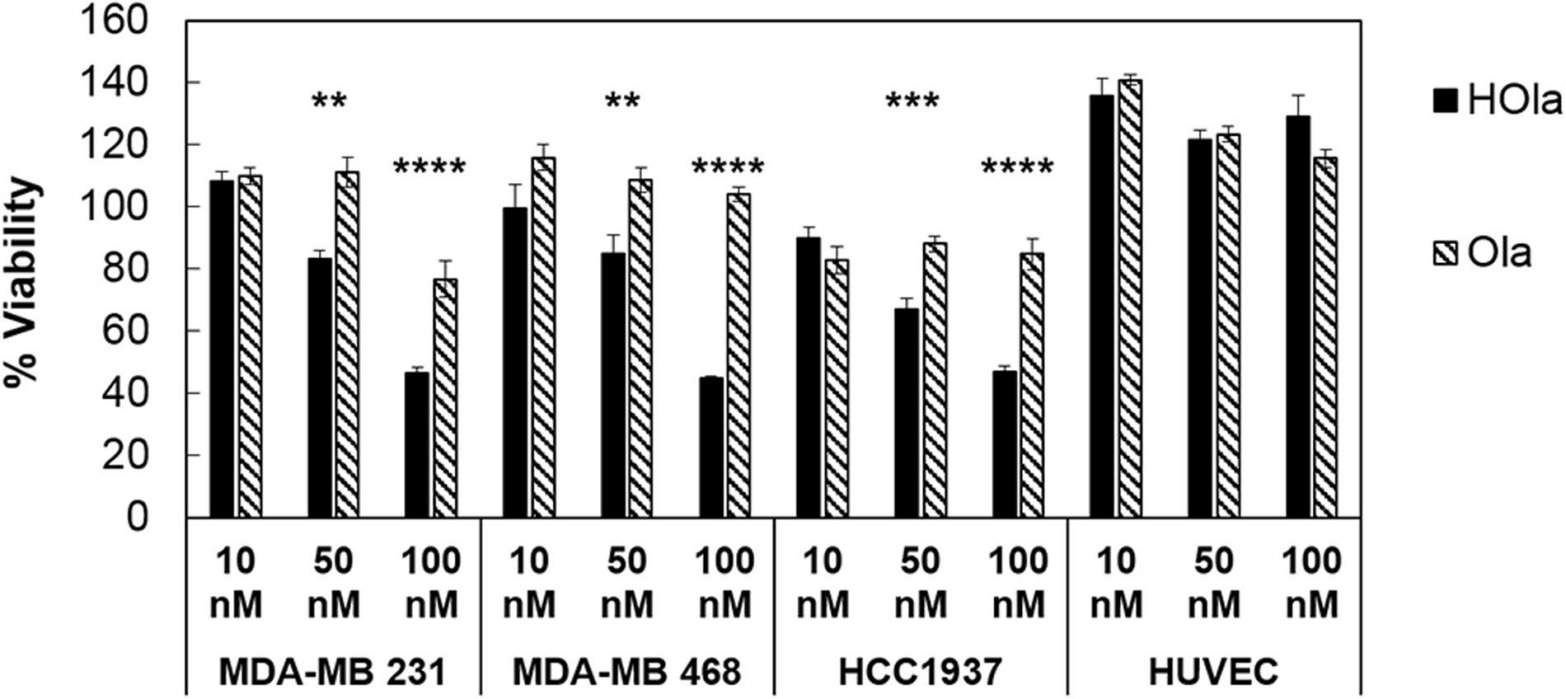
Viability of TNBC cells treated with Ola or HOla nanoparticles. HCC1937, MDA-MB 231, MDA-MB 468 and HUVEC cells were treated with 10, 50 and 100 nM Ola or HOla for 72 h. Viability was assessed by measuring the conversion of MTS into formazan. Reported values are the mean of six replicates ± s.e., normalized on cell proliferation of untreated cells, respectively. Statistical significance of HOla *vs*. free drug, **P < 0.005; ***P < 0.0005; ****P < 0.0001 (Student’s *t*-test).

HOla antitumor efficacy was compared to that of Ola. HUVEC and TNBC cells were separately treated for 72 h with increasing amounts of HOla in a drug concentration range corresponding to about 1000-fold less than the reported effective dosage of free Ola in the same cell lines (Supplementary Fig. 4). The percentage of viable cells was assessed by MTS assay on samples treated with 10, 50 and 100 nM Ola or HOla and normalized toward untreated control cells. TNBC cell proliferation was not affected by treatment with 10 and 50 nM of Ola in all samples, and only slightly decreased at 100 nM of Ola in MDA-MB 231 (Fig. 4). This is consistent with the micromolar IC_50_ values reported for Ola in MDA-MB 231, MDA-MB 468 and HCC1937 (Supplementary Fig. 4). Treatment with 10 nM of HOla did not cause any significant reduction in cancer cell proliferation. However, a notable decrease in cell viability was observed upon treatment with 50-to-100 nM of HOla drug (Fig. 4) (HOla *vs*. Ola, **P < 0.005; ***P < 0.0005; ****P < 0.0001 (Student’s *t*-test)). Interestingly, the substantial decrease in cell proliferation upon nanomolar concentrations of HOla treatment revealed remarkable differences in the antiproliferative potential of free and nanoformulated drug (Fig. 4). HOla exhibited a 1000-fold increase in efficiency toward tumor cell proliferation as compared to Ola (Fig. 4 and Supplementary Fig. 4). To exclude the toxic contribution of HFn, TNBC cells were also treated with equal amounts of void HFn. Results displayed in Supplementary Fig. 5 demonstrated the safe profile of void HFn nanocages. Of note, the proliferation of HUVEC cells was not affected by the treatment with 10, 50 and 100 nM HOla or Ola (Fig. 4). This piece of data indicated that the nanodrug does not affect the viability of healthy cells, although HFn could bind these cells.

### The treatment with HOla determines cell cycle arrest in G2/M phase

**Figure 5:**
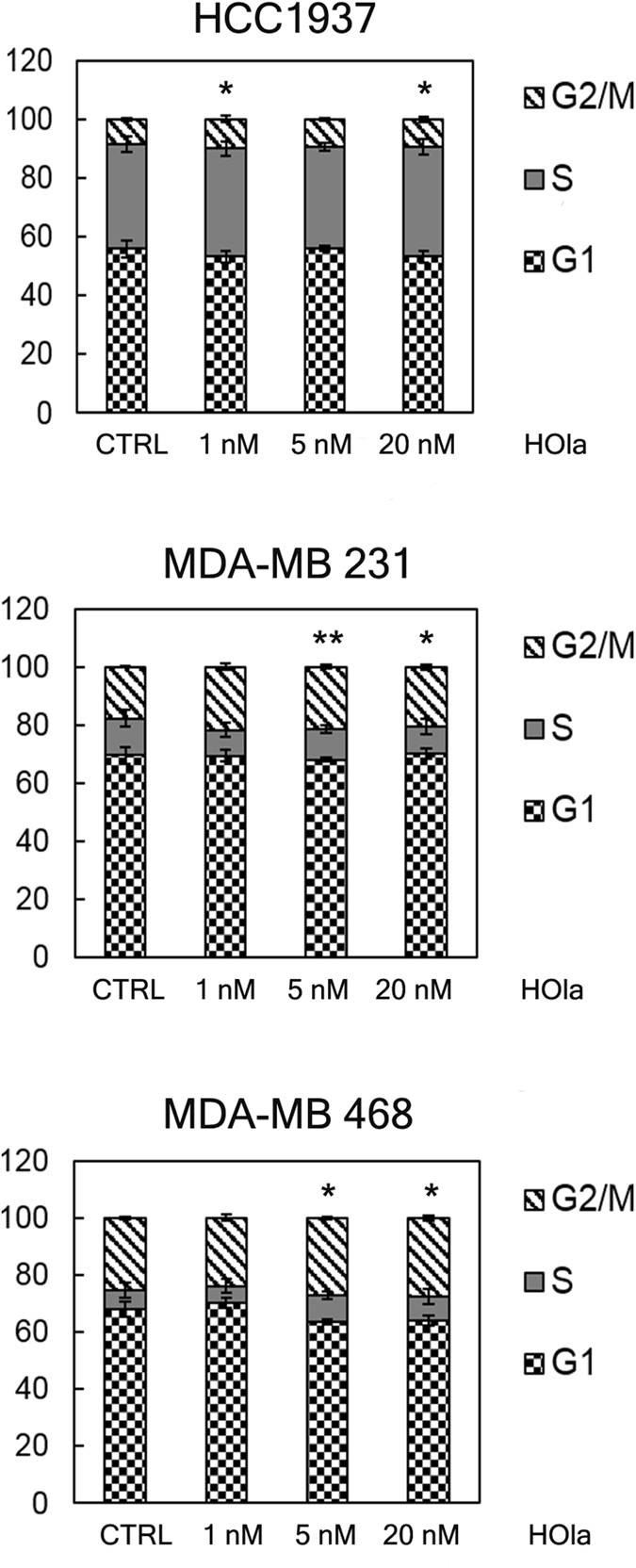
Treatment with HOla causes a cell cycle arrest in G2/M phase. HCC1937, MDA-MB 231 and MDA-MB 468 cells were incubated at 37 °C for 24 h with 1, 5 and 20 nM of HOla. Then cells were processed for flow cytometry and stained with Propidium Iodide. Untreated cells have been used as controls. Graphs represented the mean percentage of events in G1, S and G2/M phase, respectively ± s.e. (n = 3). Statistical significance of HOla *vs*. CTRL, *P < 0.05; **P < 0.01 (Student’s *t*-test).

In the attempt to investigate the bases of the higher antiproliferative activity of HOla compared to Ola, we assessed the effect of nanoformulation (1, 5 and 20 nM HOla) on the cell cycle of cultured TNBC cells (Fig. 5). Statistically significant arrest in G2/M transition was observed in TNBC cells after 24 h treatment with 20 nM HOla (HOla *vs*. CTRL, P < 0.05 (Student’s *t*-test)). Cell cycle arrest in G2/M phase was assessed upon treatment with Ola after 72 h of incubation, using different amounts of drug for each cell line depending on their drug sensitivity^26^ (HCC1937 and MDA-MB 468 cells were treated with 1, 5 and 10 µM Ola, while MDA-MB 231 cells with 10, 50 and 100 µM, according to their IC_50_) (Supplementary Fig. 6). Notably, HOla proved to be 1000-fold more effective and much faster compared to Ola. This result further corroborated the hypothesis that HFn strongly enhances the intracellular drug efficacy without changing its mechanism of action.

### HOla increases PARP-1 cleavage and DNA damage

**Figure 6:**
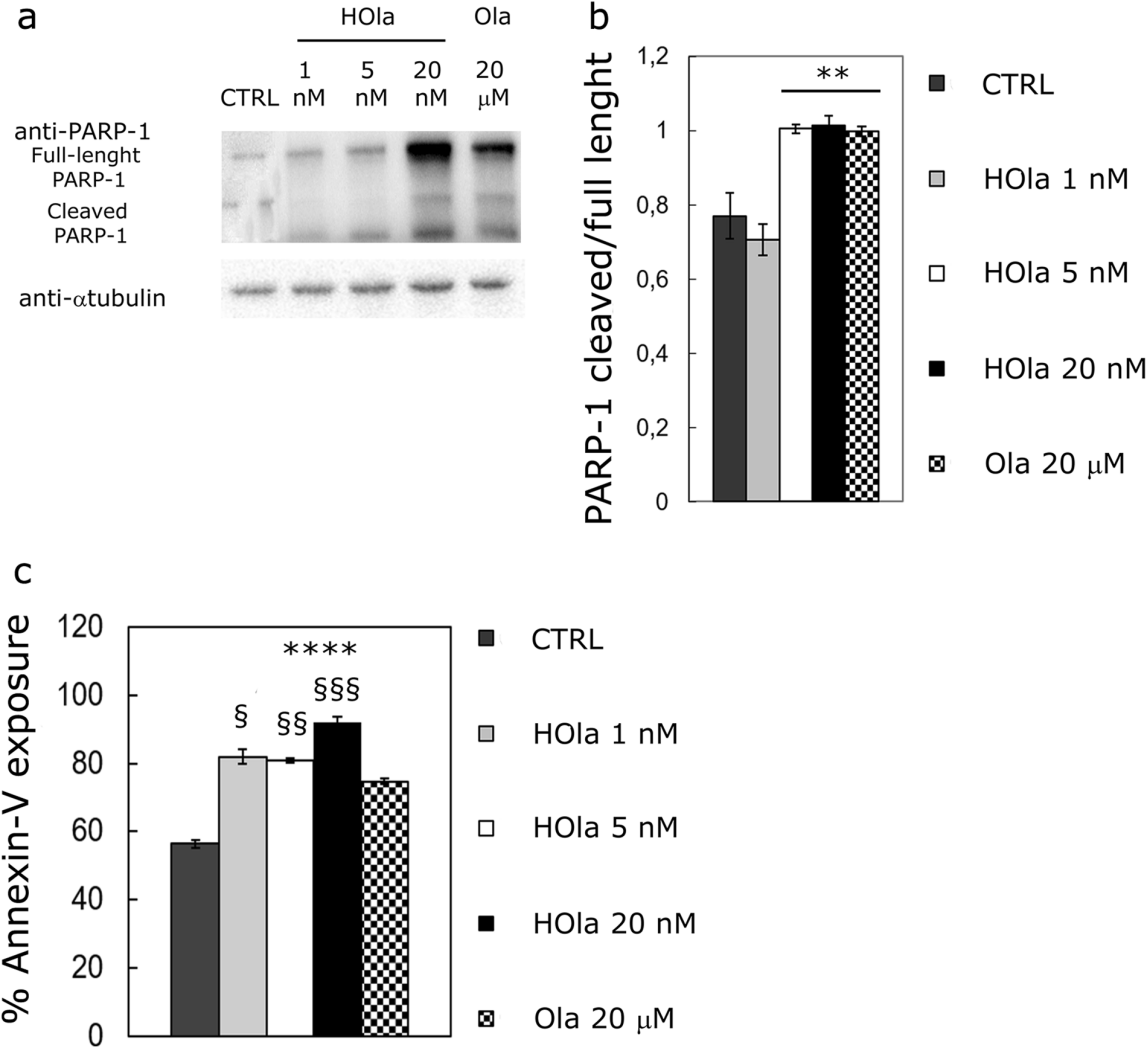
HOla is efficient in mediating PARP-1 cleavage due to Olaparib inhibition in TNBC cells. (**a**) HCC1937 were treated with increasing concentration of nanoformulated Ola (HOla; 1, 5 and 20 nM) in the presence of 5% FBS supplemented RPMI1640 medium for 72 h. As positive control, cells treated with 20 µM Ola were used. At the end of incubation, cells were lysed and subjected to western blot analysis. (**b**) Relative amounts of cleaved PARP-1 were quantified by densitometry using ImageJ Software. Reported values are the mean of 6 samples ± s.e. Statistical significance *vs*. CTRL, **P < 0.01 (Student’s *t*-test). (**c**) Cell death assay of HCC1937 cells treated with increasing concentration of nanoformulated Ola (HOla; 1, 5 and 20 nM) in the presence of 5% FBS supplemented RPMI1640 medium for 72 h. As positive control, cells treated with 20 µM Ola were used, while untreated cells were used as negative control (untreated). Reported values represent the mean ± s. e. of three replicates. (Statistical significance *vs*. CTRL, ***P < 0.0005; *vs*. Ola ^§^P < 0.05, ^§§^P < 0.005 and ^§§§^P < 0.0005).

Next, we assessed the capability of HOla to induce DNA damage and PARP cleavage in TNBC BRCA1 mutant cells. HCC1937 were incubated with HOla (1, 5 and 20 nM) or Ola (20 µM) for 72 h at 37 °C in starvation medium. Then, cells were lysed and subjected to gel electrophoresis and western blot to evaluate PARP-1 cleavage and γ-H2AX formation. Results in Fig. 6a and b display the dose-dependent increase in PARP-1 cleaved fraction (Statistical significance *vs*. CTRL, P < 0.01 (Student’s *t*-test)), which is strictly related to the Ola-mediated inhibitory effect on PARP-1. According to the results obtained by the proliferation experiments, HOla exhibited a 1000-fold increase in efficacy compared to Ola: cleaved *vs*. total PARP-1 ratio at 5–20 nM HOla was comparable to that obtained with 20 µM Ola. The proteolytic cleavage of PARP-1 was combined with the induction of apoptosis, as demonstrated by Annexin V exposure (Fig. 6c) (Statistical significance *vs*. CTRL, ***P < 0.0005; *vs*. Ola ^§^P < 0.05, ^§§^P < 0.005 and ^§§§^P < 0.0005).

**Figure 7:**
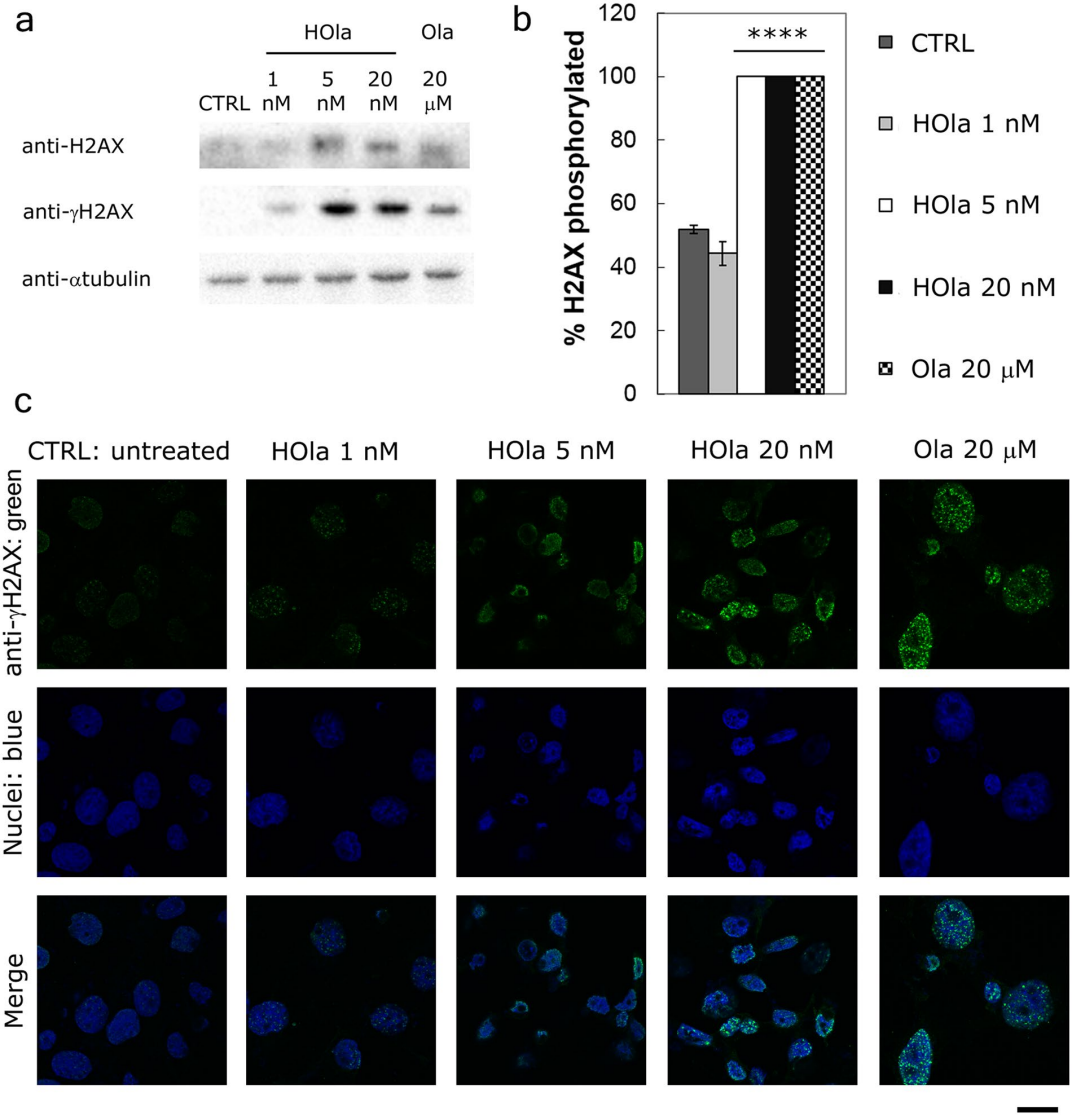
HFn increases Ola propensity to induce DNA damage in TNBC cells. (**a**) HCC1937 were treated with increasing concentration of nanoformulated Ola (HOla; 1, 5 and 20 nM) in the presence of 5% FBS supplemented RPMI1640 medium for 72 h. As a positive control, cells treated with 20 µM Ola were used. At the end of incubation, cells were lysed and subjected to western blot analysis. (**b**) The percentage of phosphorylated histone H2AX (γH2AX) were quantified by densitometry using ImageJ Software. Reported values are the mean of 6 samples ± s.e. Statistical significance *vs*. CTRL, ****P < 0.0001 (Student’s *t*-test). (**c**) Confocal microscopy images of HCC1937 cells treated with increasing concentration of nanoformulated Ola (HOla; 1, 5 and 20 nM) in the presence of 5% FBS supplemented RPMI1640 medium for 72 h. As positive control, cells treated with 20 µM Ola were used, while untreated cells were used as negative control (untreated). Nuclei were stained with DAPI (blue). DNA damage was recognized with anti-γH2AX antibody and labeled with an anti-rabbit secondary antibody conjugated with Alexa Fluor 488 (green; Thermo Fischer Scientific). Scale bar: 10 µm.

The capability of HOla to induce DNA damage was confirmed by formation of nuclear γ-H2AX. Western blot analysis and confocal microscopy imaging of HCC1937 cells treated for 72 h with increasing concentration of HOla (1, 5 and 20 nM) and Ola (20 µM) revealed the efficacy of nanocaged Ola in inducing the phosphorylation of histone H2AX (γH2AX) (Fig. 7) (Statistical significance *vs*. CTRL, P < 0.0001 (Student’s *t*-test)).

### HFn mediates nuclear delivery of Ola

**Figure 8:**
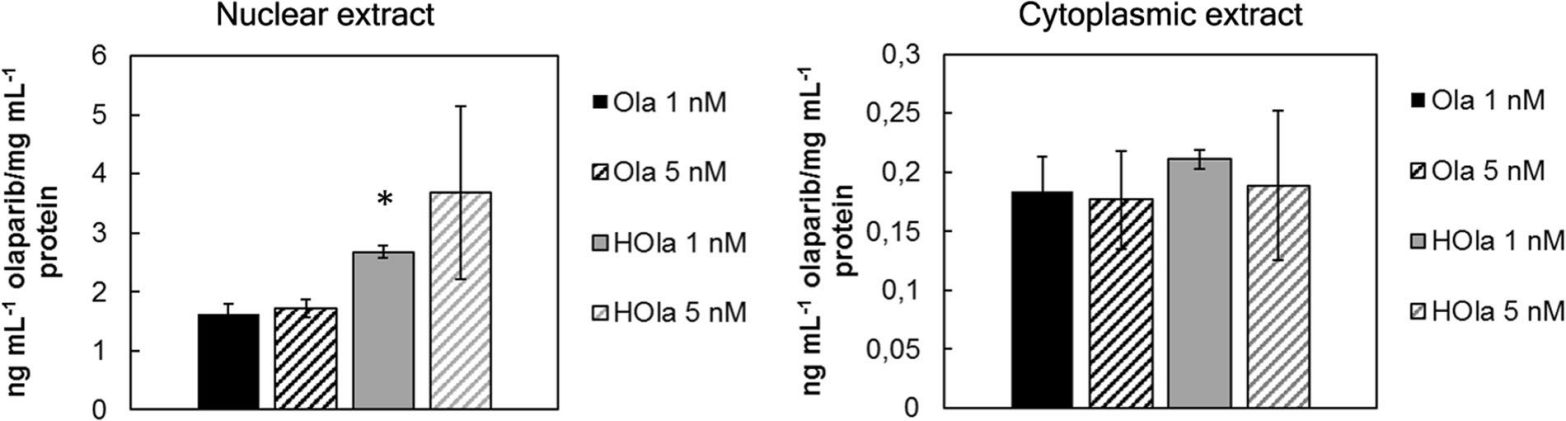
HOla promotes the drug transport into the nuclear compartment. HCC1937 were treated with increasing concentration of Ola or HOla (1 and 5 nM) for 72 h. At the end of incubation, cells were collected by tripsinization and subjected to cell fractionation to obtain crude nuclei and cytoplasmic extract. The protein content of each fraction was assessed by Bradford assay and used for normalization. Samples were processed for HPLC-MS quantification of Ola. Reported values are the mean of 2 samples ± s.e. Statistical significance HOla *vs*. Ola *P < 0.05 (Student’s *t*-test).

To explain the increased efficacy of Ola upon HFn nanoformulation, we hypothesized a positive contribution of HFn in drug delivery. Indeed, previous studies have demonstrated that HFn could function as a “Trojan horse”, by transporting cytotoxic drugs directly inside the nuclear compartment through a self-triggered mechanism of translocation, thus improving drugs’ activity and circumventing the mechanisms of multidrug resistance held by TNBC cells^21, 22^. Therefore, to investigate whether HFn could play an active role in the nuclear delivery of Ola, we treated the cells with 1 or 5 nM Ola or HOla and measured the amount of drug inside the nuclear compartment after 24 h. Results reported in Fig. 8 show that HFn did not affect Ola delivery in the cytosol, while it played a crucial role in improving its nuclear accumulation (Statistical significance HOla *vs*. Ola, P < 0.05 (Student’s *t*-test)), thus providing a rationale for the exponential increase in antitumor efficacy of HOla compared to Ola.

## Discussion

Olaparib has been recently approved in clinical practice for treatment of BC with germline mutations, including BRCA-mutated TNBC. Some phase I and II clinical trials have demonstrated inhibition of PARP as a smart strategy to chemosensitize malignant cells. By preventing DNA recovery by BER, PARP inhibitors lead to enhanced susceptibility to cytotoxic chemotherapy or, in the best cases, PARP inhibition itself could induce cell death in those cancers defective for HR mechanism^10–12, 27, 28^. Clinical data were initially encouraging for BRCA-mutated cancers^10^. A similar success was expected also for sporadic BRCA-wt TNBC treated with Ola, since various basal-like breast cancers are characterized by defects in DNA repair pathways. Hence, researchers have started to exploit the intrinsic “BRCAness” of TNBC^29^. Unfortunately, a relevant phase II clinical trial was conducted on both BRCA-mutated and sporadic TNBC patients treated with Ola, but results were discouraging, mainly in case of wt BRCA TNBCs, with no significant clinical response in sporadic breast cancers^12^.

Here, a novel nanodrug consisting of H-Ferritin nanoparticle loaded with Ola (HOla) was developed, tested and compared to free Ola for *in vitro* efficacy on three different TNBC cell lines. The rationale of our study was an optimization of the sub-cellular delivery of Ola, obtained by encapsulation of the drug into nuclear-targeted HFn nanocarrier. By pushing the drug into cancer nuclei, HOla was expected to enhance the anticancer potential in both BRCA-mutated and sporadic TNBC, by exploiting TNBC BRCAness.

HOla showed a remarkable anticancer activity as compared to Ola in all TNBC lines, with maximal differences at 100 nM (Fig. 4). While wt BRCA-TNBC cells (MDA MB-468 and MDA-MB-231) were poorly sensitive to free Ola, exhibiting global sustained viability even upon treatment with 100 nM, they surprisingly responded to equal doses of HOla, showing a marked decrease in viability down to 40%. In BRCA-mutated HCC1937 cells, Ola showed irregular anticancer activity in accordance with the reported clinical scenario. By contrast, HOla demonstrated increased dose-dependent cytotoxicity also in this TNBC subset. The negligible toxicity of void HFn nanocages (Supplementary Fig. 5), together with the conserved viability recovered in HUVEC cells treated with HOla (Fig. 4), reasonably excluded a contribution of the nanocarrier in the cytotoxic effect. These findings suggest that monotherapy based on HOla could provide potent anticancer efficacy both in BRCA-mutated and wt BRCA TNBC. A possible explanation for HOla efficacy in wt BRCA TNBC cells could reside in the genomic instability and increased cytogenetic aberrations that are associated to PARP inhibition even in HR-proficient cells^30^. Considering that many TNBCs exhibit various genomic alterations and features of “BRCAness”, it is not surprising that strong cytotoxicity of Ola can be achieved at certain dosages even in absence of BRCA mutations. Conversely, the anticancer efficacy of Ola in BRCA-mutated TNBC was expected based on the clinical literature, due to the enhanced synthetic lethality exploited by anti-PARP treatment. However, also under this setting, a significant improvement was observed with HOla, thus confirming the strong relevance of a proper nuclear delivery as a key for successful anti-PARP therapy. These data suggest that HFn-mediated nanodelivery of Ola could have a major impact also in the treatment of BRCA-mutated TNBCs.

The encapsulation of Ola in HFn nanocages offers a unique evolution in the drug aptitude to address human cancer cells and be internalized therein. HFn is actively cross-recognized by TfR1, a receptor involved in the regulation of intracellular iron. Under physiological conditions, TfR1 is ubiquitously expressed at low levels and binds transferrin^26, 31^. Interestingly, TfR1 gene is upregulated in most primary and metastatic cancer cells because higher amount of iron is required to support cancer cell proliferation. Therefore, TfR1 has been suggested as a potential target for malignant cells^31^. In addition, TfR1 expression has been proposed as a prognostic predictor, since it is related both to tumor stage and cancer progression^32^. As confirmed by flow cytometry, TNBC cell lines used in this study showed marked overexpression of TfR1 (Supplementary Fig. 1), and, as a consequence, a huge extent of HFn binding (Fig. 1). The HFn recognition of TfR1 triggered HFn internalization in TNBC cells, with prompt transfer from the membrane to the cytosolic interspace, and than to the nucleus when triggered by DNA damaging events^33^.

We have previously established that HFn is able to translocate into the nucleus and to promote the nuclear accumulation of DNA-damaging loaded drugs^22^. The same phenomenon is likely reproduced by HOla. Ola administration induced a DNA-damaging event that triggered the recruitment of ferritin inside the nuclear compartment (Fig. 2). Following this mechanism, HOla was massively delivered into the nuclear space where it promoted a targeted release of Ola on PARP system, enhancing the impairment of DNA repair and leading to cell death. After 72 h of incubation with HOla, the residual proportion of cytosolic drug was negligible, as almost the entire amount of drug was transferred into the nuclear compartment (Fig. 8). A maximum of 3 ng/mL Ola per mg/mL protein could be internalized into the nucleus even at increasing HOla concentrations, probably due to achieved saturation of the nuclear delivery system. Notably, 1 nM HOla was sufficient to reach this maximum threshold of Ola concentration in the nucleus.

All the TNBC cell lines used in this study globally showed a significant increase in anticancer efficacy by HOla. Theoretically, altered response to HOla could be observed in other TNBC cell lines, since HFn nuclear translocation could be differently effective. Moreover, different TfR1 expression levels could reduce or vary the efficacy of HOla. Further studies are needed to exactly disclose the incidence of these two parameters; however, our results support the therapeutic success of HOla in a relevant panel of TNBCs. Confocal microscopy demonstrated a prompt nuclear translocation of HFn in all TNBC cell lines (Fig. 2), suggesting that the nuclear delivery could be similarly effective in both BRCA-mutated and sporadic TNBCs. Moreover, HOla showed a greater anticancer efficacy, as compared to free Ola, also in MDA-MB 468 cells, which displayed high over-expression of TfR1 but low binding with HFn. Further confirmation of the higher efficacy of HOla was obtained investigating the PARP inhibition: the cleaved/full length PARP ratio was higher with HOla than with Ola (Fig. 6). The HOla-mediated inhibition of DNA repair was at least three orders higher than that induced by Ola, as demonstrated by the percentage of phosphorylated histone H2AX, here chosen as a marker of DNA damage (Fig. 7)^34^. As a consequence, cytotoxicity of HOla was mainly elicited in the G2/M phase, where a checkpoint is committed to arrest the cell cycle when DNA damage is not repaired (Fig. 5)^35^. Notably, the increased cleavage of PARP-1 was dependent on Ola concentration, and HOla improved drug efficacy in comparison to Ola alone. Western blot data reported that this effect was also associated to an increase in the amount of total PARP-1. A physiological cellular response to Ola is known to take place in HCC1937 cells: Ola-mediated PARP-1 cleavage is sensed by cells and stimulates the increased expression of PARP-1 in the attempt to maintain correct levels of full length PARP-1^36^. HOla is not able to circumvent this issue, since the over-expression of PARP-1 in response to the increase of PARP-1 cleavage is a physiological mechanism.

Another crucial advantage supporting the potential of HOla for TNBC treatment resides in its safety towards healthy cells. Excellent viability was observed in HUVEC cells treated with HOla despite high expression of TfR1, consistent with Ola ineffectiveness toward healthy cells (Fig. 4).

In conclusion, this is the first report demonstrating the effectiveness of nanoformulated Ola used as a cytotoxic monotherapy against TNBC. Recently, Ola has been loaded into PLGA nanoparticles, tested on lung and breast cancer cells as a chemosensitizer and compared to wortmanin, another chemosensitizer based on PI3-kinase inhibition^37^. However, while nanoformulated wortmanin showed a greater efficacy as a chemosensitizer compared to the drug alone, nanoformulated Ola demonstrated much less consistent results, particularly if conjugated to etoposide or docetaxel^37^. Ola is attracting growing interest for TNBC clinical treatment, although results from clinical trials have been controversial so far. The conventional indication for Ola is chemosensitization of BRCA-mutated TNBC to cytotoxic chemotherapy such as cisplatin, while Ola is generally not administered as a monotherapy nor used for BRCA wt cancers^27, 28^. However, achieving PARP inhibition also in BRCA wt TNBC cells with features of “BRCAness” is attracting attention among clinicians. A few trials aiming at exploring the TNBC response to Ola in relation to the genetic profile and the protein expression of the tumor are ongoing, and hopefully will identify useful predictors of clinical response to the drug^38, 39^. Our findings suggest that HFn-mediated nuclear delivery of Ola into TNBC cells could strongly enhance PARP-inhibition activity. It is plausible that HOla itself could provide enough cytotoxic activity without the need of additional drugs on both BRCA-mutated and non-mutated TNBCs. These findings are even more relevant considering the low IC_50_ of HOla and the biocompatibility profile of H-Ferritin. Further studies will be required to confirm the efficacy of HOla *in vivo* as a promising therapeutic option for TNBC.

## Methods

### HFn production

HFn nanocages were produced in *E. coli* by DNA recombinant technology and purified as previously described^22^. HFn was labelled with fluoresceine isothiocyanate (FITC) for interaction studies with TNBC cell lines.

### HFn loading with Olaparib

HOla was prepared using two different procedures: i) the disassembly/reassembly method^22^ and ii) the drug complexation with Cu(II)^24^. i) HFn nanoparticles (2 mg) were disassembled in 1.5 mL of 0.15 M NaCl by raising the pH to 12. Then, Olaparib powder (0.5 mg; Alsachim, C3734) previously solubilized by 1 h of sonication (Bransonic 12, Branson) in NaOH 0.1 M (0.5 mL) was added to the solution of disassembled HFn and incubated for 10 minutes at room temperature (RT). Then, the pH value was lowered to 7.5 using 0.1 M HCl. The resulting solution was stirred at RT for 2 h in order to favor the nanoparticle refolding. The solution was concentrated through a 100 kDa Amicon filter (Millipore) and loaded to Zeba™ Spin Desalting Column (Thermo Fischer Scientific) to remove free Ola. ii) Olaparib powder, solubilized as previously described, was incubated for 10 min at RT with 10 mM CuSO_4_ obtaining a Cu(II)-Ola complex. The complexed drug was added to HFn (2 mg) and incubated 2 h at RT. HOla was separated from free Ola by a gel filtration to Sephadex G-25 column. The amount of HFn in HOla sample was determined assessing the protein content by Bradford assay, while the amount of encapsulated Ola was determined by quantitative MS analysis, as described below.

### Ola quantification

The quantification of Ola was performed by liquid chromatography tandem mass spectrometry (LC–MS/MS) method. For quantification of Ola encapsulated in HFn nanocages, 100 µL of cold acetonitrile were added to 50 µL of HOla solution to disassemble the nanoparticles; then, samples were centrifuged 10 min at 14000 rpm to precipitate HFn. Obtained samples were spiked with Ola-*d8* as internal standard (Alsachim, Cat: C3684, IS), diluted with water/acetonitrile 95/5 and directly injected for the analysis. To study *in vitro* release of Ola, samples were treated as follow: 500 µL of samples were spiked with IS, and a liquid-liquid extraction was performed with ethyl acetate. Nuclear and cytoplasm extracts were used to quantify Ola subcellular accumulation, 100 µL of samples were spiked with IS, 200 µL of cold acetonitrile was added and the supernatant was separated by 10 min centrifugation (14000 rpm) and evaporated under vacuum. Obtained samples were dissolved in 50 µL of water/acetonitrile 50/50 and analysed. All samples were extracted and analyzed in triplicate. The liquid chromatography system was composed by a Dionex Ultimate 3000 Rapid Separation LC system (DionexThermo Fischer, Rodano Milanese, Italy). Mass analyses were performed on a ABSciex 4000 Q-trap LC-MS/MS system (AB Sciex, FosterCity, CA, USA). Ionization of analytes was performed using electrospray ion source (ESI) in positive mode. Separation of the analytes was carried out on a Phenomenex Gemini C18 column (150 mm × 2 mm i.d. 3). Calculation of Ola concentrations in biological samples was performed on three different calibration curves prepared in the same conditions of the samples described before: 0, 2–10 ng/mL for liquid-liquid extraction, 0, 5–10 ng/mL for the nuclear and cytoplasm extracts and 25–750 ng/mL for loading samples.

### HOla characterization by Dynamic Light Scattering and ζ-potential

The HFn hydrodynamic diameter and ζ-potential were analyzed on a Zetasizer Nano ZS ZEN3600 (Malvern Instruments Ltd) operating at light source wavelength of 633 nm and a fixed angle of 173°. For Dynamic Light Scattering (DLS) analysis, the purified samples were diluted in PBS buffer, pH 7.2. For ζ-potential, the samples were diluted in milli-Q water (Millipore). The results were expressed as mean ± standard deviation of three measurements.

### Transmission Electron Microscopy

A drop of HFn suspension was placed on the Formvar net and dried at RT. Then, the net was washed twice with milli-Q water, stained with uranil-acetate 1% for 30 min at RT and dried over night at RT. Samples were examined by Transmission Electron Microscopy (Tecnai Siprit, FEI).

### Kinetics of Ola spontaneous release *in vitro*

HOla was stored in a dialysis device and kept in a PBS bath at 37 °C for three days. At predetermined time points (15 min, 30 min, 1 h, 2 h, 3 h, 4 h, 6 h, 24 h, 48 h, 72 h), the amount of released drug was quantified by HPLC coupled mass spectrometry and the buffer was replaced after each measurement in order to maintain sink condition.

### Cell cultures

MDA-MB 468 cells were cultured in Dulbecco’s Modified Eagle’s Medium High Glucose (DMEM), supplemented with 10% fetal bovine serum (FBS), L-glutamine (2 mM), penicillin (50 UI/mL) and streptomycin (50 mg/mL). HCC1937 cells were cultured in RPMI 1640 medium, supplemented with 10% FBS, L-glutamine (2 mM), penicillin (50 UI/mL) and streptomycin (50 mg/mL). MDA-MB 231 cells were cultured in MEM Medium, supplemented with 10% FBS, L-glutamine (2 mM), penicillin (50 UI/mL) and streptomycin (50 mg/mL), while HUVEC were cultured in EGM™-2 Bullet kit medium (Lonza). All cell lines grew at 37 °C and 5% CO_2_ in a humidified atmosphere and were subcultured prior to confluence using trypsin/EDTA. Cell culture medium and chemicals for cell culture were purchased from Euroclone.

### TfR1 expression

MDA-MB 231, MDA-MB 468, HCC1937 and HUVEC cells (5 × 10^5^) were immunodecorated in FACS tubes with anti-TfR1 antibody (1 µg/tube; clone ICO-92; Thermo Fischer Scientific) in Phosphate buffer (PBS), 2% Bovine Serum Albumin (BSA; Sigma) and 2% goat serum (Euroclone) for 30 min at RT. Then, cells were washed thrice with PBS and immunodecorated with Alexa Fluor 488 goat anti-mouse secondary antibody (1 µL/tube; Thermo Fischer Scientific) in PBS, 2% BSA and 2% goat serum for 30 min at RT. After three washes with PBS cells were analyzed by CytoFLEX flow cytometer (Beckman Coulter). 20,000 events were acquired for each analysis, after gating on viable cells and on singlets. A sample of cells immunodecorated with the secondary antibody only was used to set the region of positivity.

### Ferritin heavy chain expression

About 5 × 10^5^ cells were lysed with 200 µL lysis buffer (20 mM Tris HCl pH 7.6, 150 mM NaCl, 1 mM EDTA, 1% Triton X-100, 1% glycerol, 1 mM Na_3_VO_4_, 10 mM NaF, Protease Inhibitor Cocktail, 1 mM PMSF). Protein content in lysate was quantified using the Coomassie Plus Protein Assay Reagent (Thermo Fisher Scientific) with Bovine Serum Albumin (BSA) as standard protein. Approximately 30 µg of protein from each sample were separated by SDS-PAGE and then transferred onto PVDF membrane. The membrane was blocked in 5% BSA in TBS with 0.1% Tween 20 for 1 h. The membrane was incubated 2 h at RT with rabbit-monoclonal antibody against Ferritin heavy chain (Abcam, Ab7332) at 1:1000 dilution and with mouse monoclonal antibody anti-α-tubulin (Sigma) at 1:1000 dilution in 5% BSA in TBS with 0.1% Tween 20. The membranes were washed three times with TBS with 0.1% Tween 20 and reacted 1 h with the secondary antibody anti-rabbit conjugated with horseradish peroxidase (1:5000; Abcam) The bound antibody was revealed using ECL star reagent (Euroclone) and the chemoluminescence signal was detected using the Chemidoc System (Biorad). Quantification has been performed with imageJ software on three samples/group and results have been expressed as mean ± s.e.

### In tube-cell binding assay at 4 °C

5 × 10^5^ cells/tube were collected and incubated for 2 h at 4 °C in blocking buffer (PBS, 0.3% BSA) supplemented with 20 and 100 µg/mL of FITC-labelled HFn. After incubation, cells were washed three times with PBS, suspended in 0.5 mL of PBS and analyzed by CytoFLEX flow cytometer (Beckman Coulter). 20,000 events were acquired for each analysis, after gating on viable cells and on singlets. A sample of untreated cells was used to set the appropriate gates.

### In plate-cell binding assay at 37 °C

MDA-MB 231, MDA-MB 468 and HCC1937 cells were seeded at a concentration of 2.5 × 10^5^ cells/well. The day after, cells were incubated 1 h at 37 °C in culture medium supplemented with 20 and 100 µg/mL of FITC-labelled HFn. After incubation, cells were washed three times with PBS, detached from the plate with trypsin/EDTA, suspended in 0.5 mL of PBS and analyzed by CytoFLEX flow cytometer (Beckman Coulter). 20,000 events were acquired for each analysis, after gating on viable cells and on singlets. A sample of untreated cells was used to set the appropriate gates.

### Competition assay

HCC1937 cells were seeded at a concentration of 5 × 10^5^ cells/well. The day after, 100 µg/mL of FITC-labelled HFn were incubated with the cells for 1 h at 37 °C with or without 5 mg of unlabelled HFn added as competitor. After incubation, cells were washed three times with PBS, detached from the plate with trypsin/EDTA, suspended in 0.5 mL of PBS and analyzed by CytoFLEX flow cytometer (Beckman Coulter). 10,000 events were acquired for each analysis, after gating on viable cells and on singlets. A sample of untreated cells was used to set the appropriate gates.

### Confocal laser scanning microscopy

Cells were cultured until sub-confluence on cover glass slides pre-coated with collagen, and incubated with FITC-labelled HFn for different time periods. To evaluate internalization, TfR1 colocalization and sub-cellular localization, 100 µg/mL of FITC-labelled HFn were incubated with cells for 15 min, 1, 3, 24 and 48 h at 37 °C. To evaluate Ola capability to trigger nuclear translocation of endogenous ferritin cells were incubated in presence of Ola 1 µM for 1, 3, and 48 h at 37 °C. After incubations, cells were washed with PBS, fixed for 5 min with 4% paraformaldehyde (Sigma) and then treated for 5 min with 0.1% Triton X-100 (Sigma). A blocking step was performed for 1 h at RT with a solution containing 2% BSA (Sigma), 2% goat serum (Euroclone) and 0.2 µg/mL DAPI (4’,6-diamino-2-phenylindole; Thermo Fischer Scientific) in PBS. Golgi apparatus, lysosomes, early and recycling endosomes were stained for 2 h at RT with Golgi marker 130 (GM-130; at a 1:100 dilution; clone 35; BD Biosciences), Cathepsin D (CatD; 1:50; clone BC011; Calbiochem), Early Endosomes Antigen-1 (EEA-1; 1:1000; clone 14; BD Biosciences) and Transferrin (Tf; 1:100; clone 5G2; Abcam) antibodies, respectively, and revealed by Alexa Fluor 546-conjugated antibody against murine IgGs (Thermo Fischer Scientific) at a 1:300 dilution by incubating for 2 h at RT in PBS, 2% BSA, 2% goat serum. Endogenous ferritin was stained with the anti-ferritin heavy chain antibody (1:2000; ab65080; Abcam) and revealed by Alexa Fluor 488-conjugated antibody against rabbit IgGs (Thermo Fischer Scientific) at a 1:300 dilution by incubating for 2 h at RT in PBS, 2% BSA, 2% goat serum. DNA damage was revealed by the anti-γH2AX antibody (1:1000; ab11174; Abcam) and Alexa Fluor 488-conjugated antibody against rabbit IgGs (Thermo Fischer Scientific) at a 1:300 dilution by incubating for 2 h at RT in PBS, 2% BSA, 2% goat serum. TfR1 colocalization was revealed with the anti-TfR1 antibody (1:200; ab84036; Abcam) and recognized by Alexa Fluor 546-conjugated antibody against rabbit IgGs (Thermo Fischer Scientific) at a 1:300 dilution by incubating for 2 h at RT in PBS, 2% BSA, 2% goat serum. Images were acquired with Leica SPE microscope confocal system equipped with laser excitation lines 405 nm, 488 nm, 514 nm and 633 nm or with Leica SP8 microscope confocal system equipped with laser excitation lines 405 nm, 488 nm, 535 nm and 633 nm. Images were acquired with 63× magnification oil immersion lenses at 1024 × 1024 or 512 × 512 pixel resolution.

### Cell viability assay

MDA-MB 231, MDA-MB 468 and HCC1937 cells were seeded on a 96 multi-well dish at the density of 5000 cells cm^−1^. Then, cells were incubated with different amounts of Ola (Ola: 10, 50, 100 nM and 10, 20, 50, 100 and 200 µM; HOla: 10, 50, 100 nM). Untreated cells were used as controls. After 72 h of treatment, cells were washed with PBS and incubated for 3 h at 37 °C with 0.1 mL of a stock solution of 3-(4,5-dimethylthiazol-2-yl)-5-(3-carboxymethoxyphenyl)-2-(4-sulfophenyl)-2H-tetrazolium (MTS) and phenazine ethosulfate (PES) previously diluted 1:10 in DMEM medium without phenol red (CellTiter 96® AQueous One Solution Reagent; Promega). Absorbance was read in a microplate reader (BioTek) using a testing wavelength of 490 nm and a reference wavelength of 620 nm. The results were normalized on viability of untreated samples and expressed as means ± s.e.

### Cell Cycle analysis

2.5 × 10^5^ cells were seeded on a 12 multi-well dish the day before. Then, cells were incubated with different amounts of HOla (1, 5, 20 nM) for 24 h in medium supplemented with FBS 5%. Untreated cells were used as negative control, while cells treated for 72 h with free Ola were used as positive controls (MDA-MB 468 and HCC1937 cells: 1, 5 and 10 µM; MDA-MB 231: 10, 50 and 100 µM). At the end of the incubation time, cells were washed twice with PBS, detached with Trypsin/EDTA solution and transferred in FACS tubes. Then, cells were fixed with cold ethanol 95% for 1 h, labelled with staining solution (PBS supplemented with 80 µg/mL Iodide Propidium (Sigma), 100 µg/mL RNAseA (Sigma) and 0.1% Triton X-100 (Sigma)) and acquired with CytoFLEX flow cytometer (Beckman Coulter).

### Evaluation of PARP-1 cleavage and histone H2AX phosphorylation

HCC1937 cells cultured in a 6-wells plate were treated for 72 h with increasing concentration of HOla (1, 5 and 20 nM) in RPMI 1640 medium supplemented with 5% FBS. Cells treated with 20 µM of free Ola were used as positive control. Negative control was represented by untreated cells. Cells were lysed with 200 µL lysis buffer (20 mM Tris HCl pH 7.6, 150 mM NaCl, 1 mM EDTA, 1% Triton X-100, 1% glycerol, 1 mM Na_3_VO_4_, 10 mM NaF, Protease Inhibitor Cocktail, 1 mM PMSF), and the protein content was quantified using the Coomassie Plus Protein Assay Reagent (Thermo Fisher Scientific) with BSA as standard protein. Approximately 30 µg of protein from each sample were separated by SDS-PAGE and transferred onto PVDF membrane. The membrane was blocked in 5% BSA in TBS with 0.1% Tween 20 for 1 h. For evaluation of PARP-1 cleavage, the membrane was incubated overnight with rabbit-monoclonal antibody against PARP-1 (clone 46D11; Cell Signalling) at 1:1000 dilution and with mouse monoclonal antibody anti-α-tubulin (Sigma) at 1:1000 dilution in 5% BSA in TBS with 0.1% Tween 20 for 1 h. To assess histone H2AX phosphorylation, the membrane was incubated overnight with rabbit-monoclonal antibody against histone H2AX (#2595; Cell Signalling) at 1:1000 dilution, phosphorylated histone H2AX (Ser139) (clone 20E3; Cell Signalling) at 1:1000 dilution, or a mouse monoclonal antibody anti-α-tubulin (Sigma) at 1:1000 dilution in 5% BSA in TBS with 0.1% Tween 20 for 1 h. The membranes were washed three times with TBS with 0.1% Tween 20 and reacted 1 h with the secondary antibody anti-rabbit conjugated with horseradish peroxidase (1:5000; Abcam) or with the secondary antibody anti-mouse conjugated with horseradish peroxidase (1:5000; Abcam), respectively. The bound antibody was revealed using ECL star reagent (Euroclone) and the chemoluminescence signal was detected using the Chemidoc System (Biorad).

### Cell death assay

HCC1937 cells seeded on a 6-well plate at 2 × 10^5^ cells/well were treated for 72 h with increasing concentration of HOla (1, 5 and 20 nM) in RPMI 1640 medium supplemented with 5% FBS. As positive control, cells treated with 20 µM of free Ola were used. Negative control was represented by untreated cells. Then, cells were collected, washed thrice with PBS and treated for FACS analysis according to Annexin V-PE-Cy5 Apoptosis Detection Kit manufacturer’s protocol (BioVision). Briefly, cells were suspended in Binding Buffer and incubated 5 min with 5 µL of Annexin V-PE-Cy5. Cells were analyzed within 1 h on CytoFLEX flow cytometer (Beckman Coulter). 20,000 events were acquired for each analysis.

### Quantification of Nuclear accumulation of Ola

HCC1937 cells were seeded in a 6-multiwell plate at 1 × 10^6^ cells/well. Cells were incubated at 37 °C with Ola or HOla (5 and 1 nM) for 24 h. At the end of incubation, cells were harvested with Trypsin/EDTA and centrifuged 5 min at 2,000 rpm. Pellets were washed twice with PBS, suspended in 1 mL of Nuclei Extraction Buffer (10 mM Hepes, pH 7.4, 320 mM Sucrose, 5 mM MgCl, 1% Triton X-100) and incubated on ice for 10 min. Then, nuclei were pelleted by 5 min centrifugation at 2,000 rpm and washed twice with 1 mL of Nuclei Wash Buffer (10 mM Hepes, pH 7.4, 320 mM Sucrose, 5 mM MgCl). Purified nuclei were processed for HPLC/MS evaluation of Ola content, as reported above. Protein content of purified nuclei was assessed by Bradford assay and used for normalizations. The purity of nuclei and cytoplasmatic fractions was checked by western blot using anti-rabbit-α-tubulin (1:1000 dilution, Sigma) and anti-rabbit-monoclonal antibody against histone H2AX (1:1000 dilution, #2595; Cell Signalling), and reported in Supplementary Fig. 7.

## Electronic supplementary material

Supplementary Information
