# Supplementary material for: H-Ferritin-nanocaged olaparib: a promising choice for both BRCA-mutated and sporadic triple negative breast cancer
Source: Sci Rep. 2017 Aug 8;7:7505. doi: 10.1038/s41598-017-07617-7 (PMC5548799; doi:10.1038/s41598-017-07617-7)
Supplement: Supplementary file 1 — Supplementary Information [file 41598_2017_7617_MOESM1_ESM.pdf]

Supporting informations

H-Ferritin-nanocaged olaparib: a promising choice for both BRCA-mutated and sporadic triple negative breast cancer

Mazzucchelli S., [1] Truffi M.,[1] Baccarini F.,[1] Beretta M.,[1] Sorrentino L.,[1] Bellini M.,[2] Rizzuto M. A.,[2] Ottria R.,[1] Ravelli A.,[3] Ciuffreda P.,[1] Prosperi D.,[2*] Corsi F.[1,4*]

[1] Dipartimento di Scienze Biomediche e Cliniche "Luigi Sacco", Università degli Studi di Milano, Milano, Italy.

[2] Dipartimento di Biotecnologie e Bioscienze, Università degli Studi di Milano-Bicocca, Milano, Italy.

[3] Dipartimento di Scienze Biomediche, Chirurgiche ed Odontoiatriche, Sezione di Tossicologia Forense, Università degli Studi di Milano, Milano, Italy.

[4] Unità di chirurgia generale ad indirizzo senologico, Istituto scientifico di Pavia, ICS Maugeri Spa SB, Pavia, Italy

*Corresponding Authors

Prof. Fabio Corsi:

phone: (+39)0250319850-9858; email: fabio.corsi@unimi.it

Prof. Davide Prosperi:

phone: (+39)0264483302; Fax: (+39)0264483565; email: davide.prosperi@unimib.it*

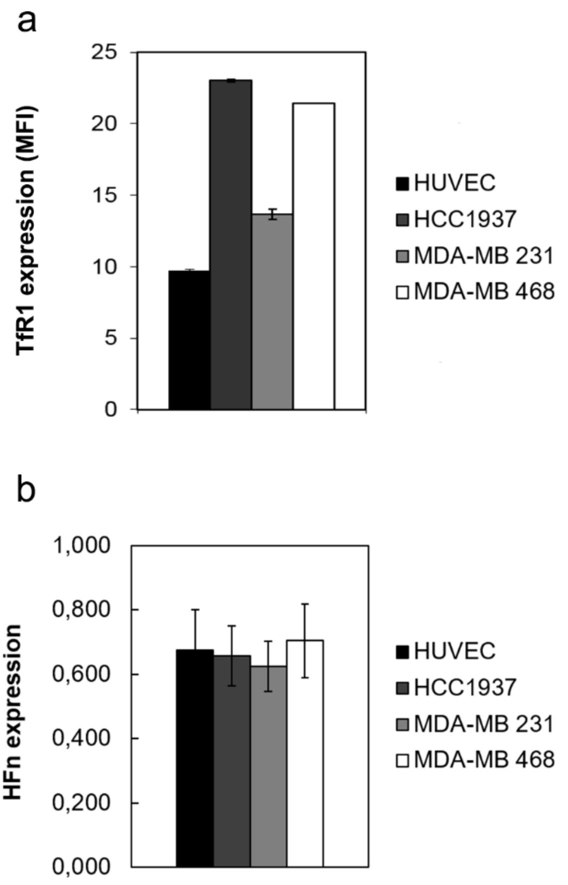

Supplementary Figure 1. Expression of TfR1 and heavy-chain ferritin in human cell lines. (**a**) Flow cytometry analysis of membrane TfR1 expression. Three human triple negative breast cancer cell lines (HCC1937, MDA-MB 468 and MDA-MB 231) and the HUVEC cells have been tested assessing their membrane TfR1 expression. Cells immunodecorated with the anti-mouse secondary antibody conjugated with AlexaFluor 488 were used to set the gate on viable cells, on singlets and the region of positivity. (**b**) Western blot quantification of endogenous ferritin heavy chain in whole cell lysates of HCC1937, MDA-MB 468, MDA-MB 231 and HUVEC cell lines.

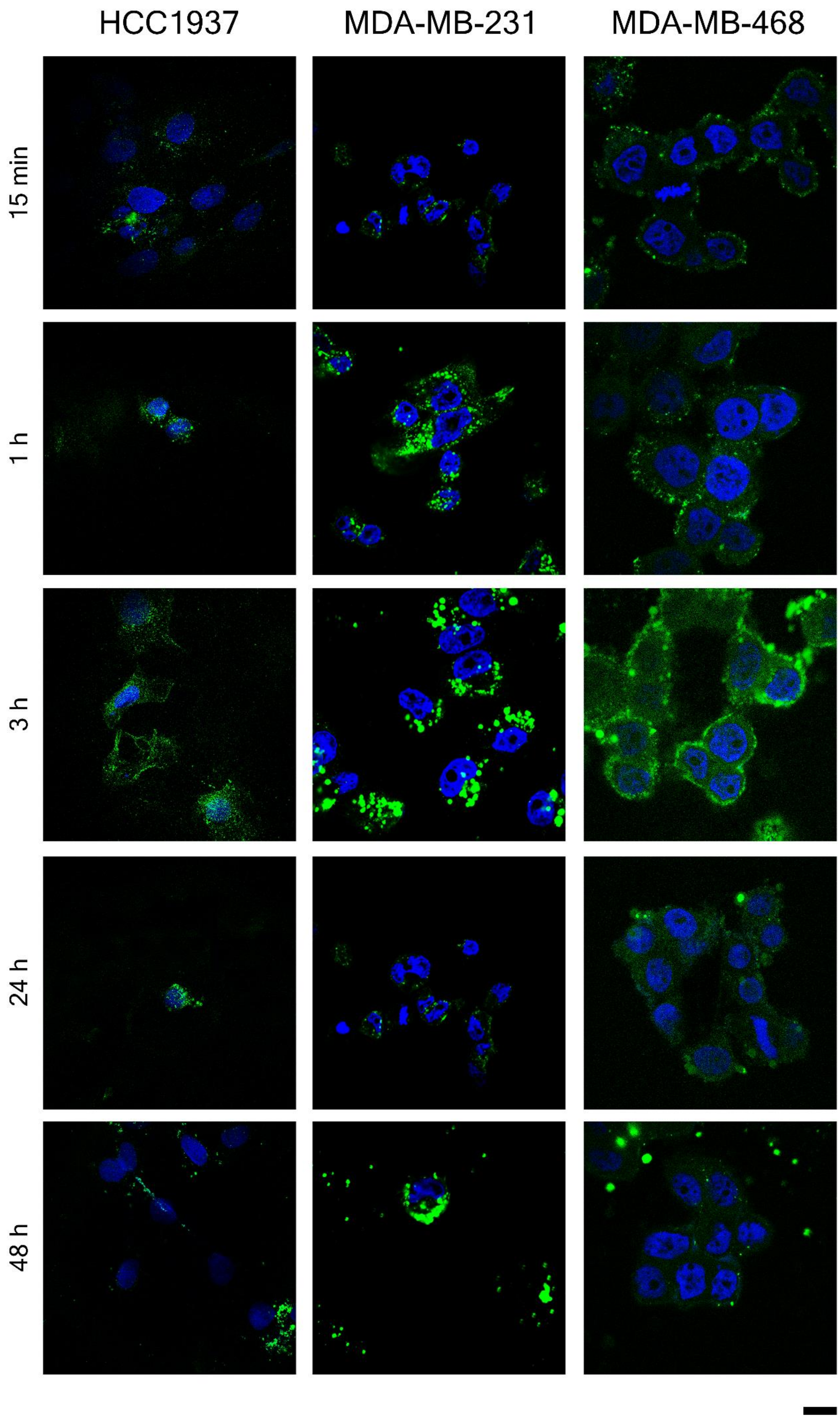
HCC1937
MDA-MB-231
MDA-MB-468
15 min
1 h
3 h
24 h
48 h

Supplementary Figure 2: Time course of internalization of HFn nanoparticles in TNBC cells. Confocal microscopy images of HCC1937, MDA-MB 231 and MDA-MB 468 cells incubated for 15 minutes, 1 h, 3 h, 24 h and 48 h at 37 °C with 100 µg/mL of FITC-labelled HFn (green). Nuclei were stained with DAPI (blue). Scale bar: 10 µm.

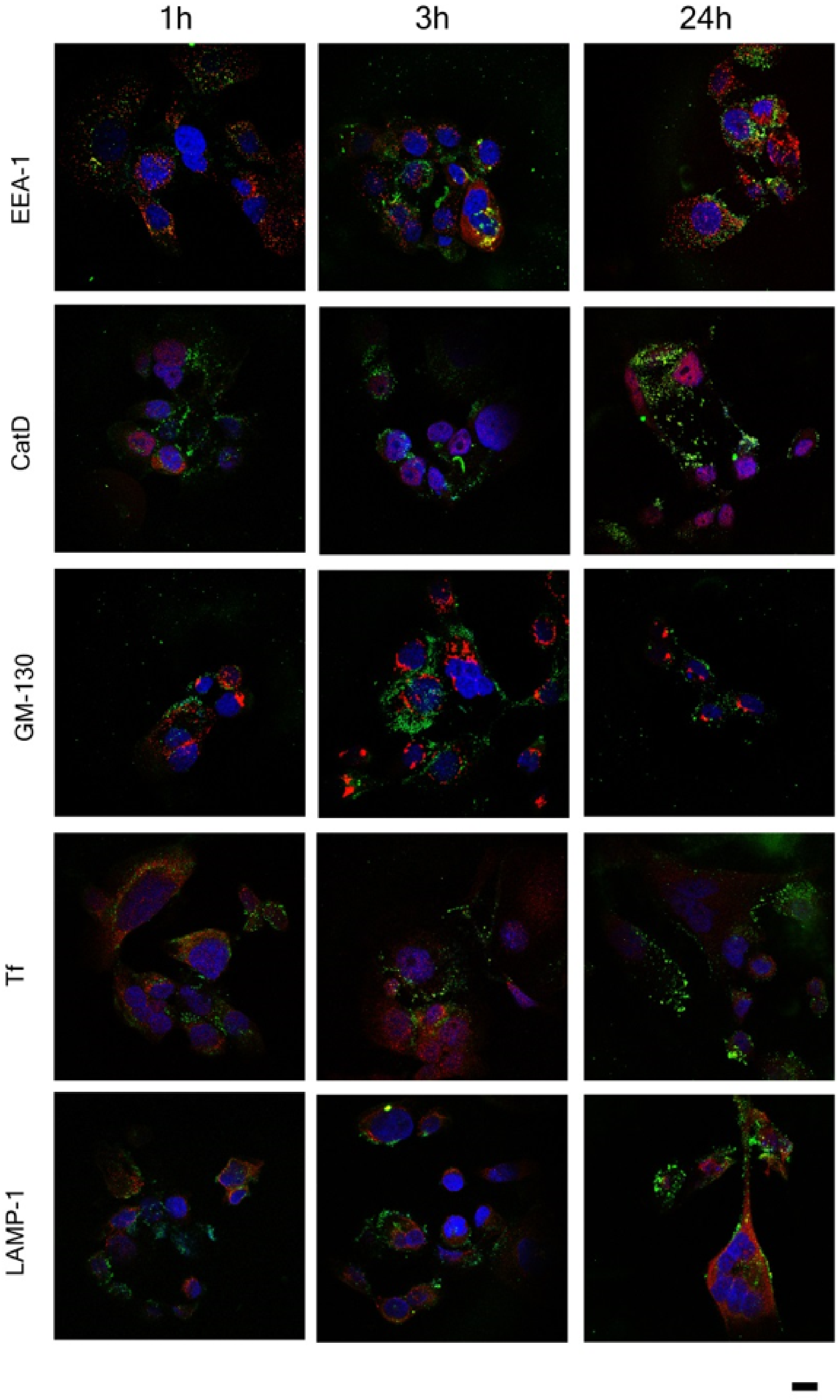

Supplementary Figure 3: Intracellular localization of HFn nanoparticles. Confocal microscopy merge images of HCC1937 cells incubated for 1, 3 and 24 at 37 °C with 100 µg/mL of FITC-labelled HFn (green). Early endosomes, lysosomes, Golgi and recycling endosomes were stained with antibodies against the early endosome marker EEA1, the lysosomal protein CatD, the Golgi marker GM130 and the recycling endosome marker Tf, respectively, and labeled with an anti-mouse secondary antibody conjugated with Alexa Fluor 546 (red). Nuclei were stained with DAPI (blue). Scale bar: 10 µm.

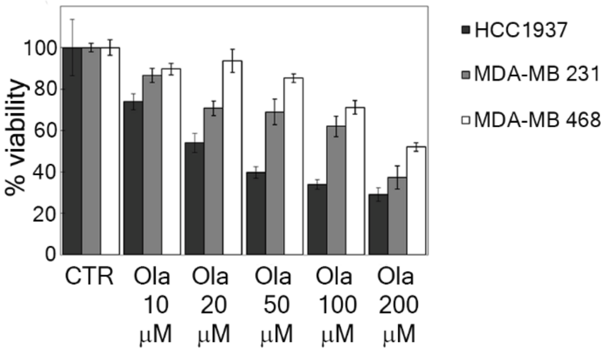

Supplementary Figure 4: Viability of TNBC cells treated with free Olaparib. HCC1937, MDA-MB 231 and MDA-MB 468 were treated with 10, 20, 50, 100 and 200 µM of Ola for 72 h. Viability was assessed by measuring the conversion of MTS into formazan. Reported values are the mean of six replicates ± s.e., normalized on the proliferation of untreated cells (CTR).

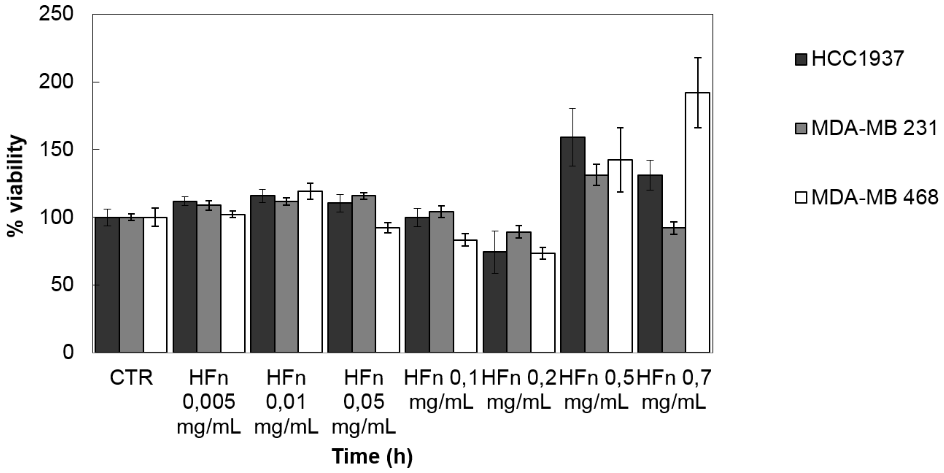

Supplementary Figure 5. Viability of TNBC cells treated with void HFn. HCC1937, MDA-MB 231 and MDA-MB 468 were treated with 0.005, 0.01, 0.05, 0.1, 0.2, 0.5 and 0.7 mg/mL of void HFn for 72 h. Viability was assessed by measuring the conversion of MTS into formazan. Reported values are the mean of six replicates ± s.e., normalized on the proliferation of untreated cells (CTR).

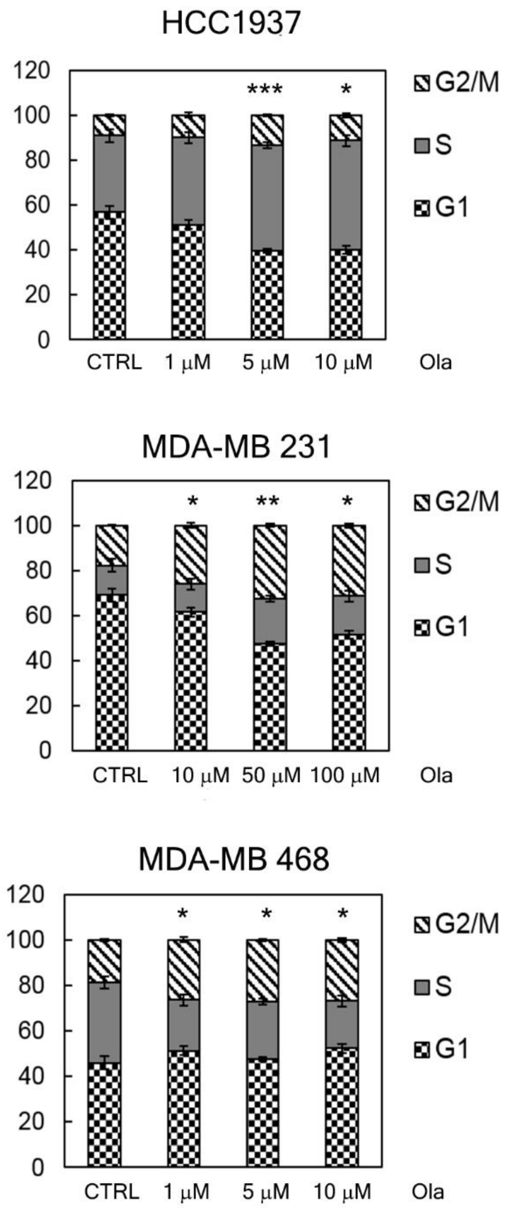

Supplementary Figure 6. Treatment with Ola causes a cell cycle arrest in G2/M phase. HCC1937 and MDA-MB 468 cells were incubated at 37 °C for 72 h with 1, 5 and 10 µM of Ola, while MDA-MB 231 cells were incubated with highest Ola concentrations (10, 50 and 100 µM). Cells were processed for flow cytometry, stained with Propidium Iodide and analyzed with Cytoflex (Beckman Coulter). Untreated cells were used as controls. Graphs represented the mean percentage of events in G1, S and G2/M phase, ± s.e. (n=3). Statistical significance of Ola vs CTRL, * P<0.05; ** P<0.01; *** P<0.001 (Student's t-test).

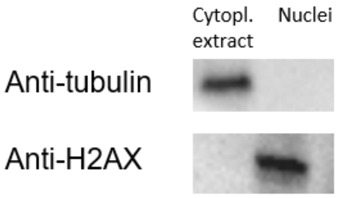

Supplementary Figure 7. Control of purity of nuclei and cytoplasmic fractions. Western blot analysis was performed on nuclei and cytoplasmic extract samples using the anti-tubulin, as marker of the cytoplasmic fraction, and the anti-histone H2AX, as nuclei marker.
